# Inhibition of Cholesteryl Ester Transfer Protein Contributes to the Protection of Ginsenoside Re Against Isoproterenol-Induced Cardiac Hypertrophy

**DOI:** 10.7759/cureus.59942

**Published:** 2024-05-09

**Authors:** Yumei Qiu, Mengdie Xie, Xiaoyun Ding, Hao Zhang, Hongming Li, Hu Wang, Tingting Li, Wei Dong, Fangqin Jiang, Xilan Tang

**Affiliations:** 1 School of Pharmacy, Jiangxi Science & Technology Normal University, Nanchang, CHN; 2 Key Laboratory of Modern Preparation of Chinese Medicine, Ministry of Education, Jiangxi University of Traditional Chinese Medicine, Nanchang, CHN; 3 Cardiovascular Pharmacology of Chinese Medicine, Jiangxi Provincial Key Laboratory of Drug Design and Evaluation, Nanchang, CHN

**Keywords:** ginsenoside re, cardiac hypertrophy, cholesteryl ester transfer protein, isoproterenol, high-density lipoprotein cholesterol

## Abstract

Background and objectives

Ginsenoside Re (Re), a protopanaxatriol-type saponin extracted from ginseng, is known to have potential cardioprotective effects; however, the mechanisms of Re in improving cardiac hypertrophy have not been fully elucidated. This study aimed to investigate the therapeutic effects and underlying mechanism of Re on isoproterenol (ISO)-induced cardiac hypertrophy *in vivo* and *in vitro*.

Methods

Rats were intraperitoneally injected with ISO 30 mg/kg thrice daily for 14 consecutive days to induce cardiac hypertrophy, and these rats were treated with atorvastatin (ATC, 20 mg/kg) or Re (20 mg/kg or 40 mg/kg) once daily for three days in advance until the end of the experiment. Heart weight index, hematoxylin and eosin staining, and hypertrophy-related fetal gene expression were measured to evaluate the effect of Re on cardiac hypertrophy *in vivo*. Meanwhile, the rat H9c2 cardiomyocyte hypertrophy model was induced by ISO 10 μM for 24 hours. Cell surface area and hypertrophy-related fetal gene expression were determined to assess the effect of Re on ISO-induced cardiomyocyte hypertrophy *in vitro*. The levels of total cholesterol (TC), triglyceride (TG), low-density lipoprotein cholesterol (LDL-C), and high-density lipoprotein cholesterol (HDL-C) in both serum and cardiomyocytes were detected by enzymatic colorimetric assays. Furthermore, we chose cholesteryl ester transfer protein (CETP) as a target to explore the influence of Re on CETP expression *in vivo* and *in vitro* through real-time polymerase chain reaction, western blot, and enzyme-linked immunosorbent assay.

Results

Intraperitoneal administration of ISO into rats resulted in increases in cross-sectional cardiomyocyte area, the ratio of heart weight to body weight, the ratio of left ventricular weight to body weight, and the ratio of right ventricular weight to body weight, as well as reactivation of fetal genes; however, treatment with Re or ATC ameliorated most of these hypertrophic responses. Similarly, Re pronouncedly alleviated ISO-induced cardiomyocyte hypertrophy, as evidenced by a decreased cell surface area and downregulation of fetal genes. Moreover, our *in vivo* and *in vitro* data revealed that Re reduced TC, TG, and LDL-C levels, and enhanced HDL-C levels. Re improved cardiac hypertrophy mainly associated with the inhibition of mRNA level and protein expression of CETP, to an extent comparable to that of the classical CETP inhibitor, anacetrapib.

Conclusions

Our research found that CETP inhibition contributes to the protection of Re against ISO-induced cardiac hypertrophy, which provides evidence for the application of Re for cardiovascular disease treatments.

## Introduction

Cardiovascular disease remains one of the leading causes of morbidity and mortality worldwide. Cardiac hypertrophy is a pivotal pathological process that exists in multiple cardiovascular diseases, such as hypertension, cardiomyopathy, and valvular disease, and is manifested as abnormal increases in mass and volume of the myocardium, enlargement of individual cardiomyocytes, and reprogramming expression of fetal genes [1,2]. Therefore, more effective therapies for cardiac hypertrophy are highly needed, which is extremely important to reduce cardiovascular risk.

Cholesteryl ester transfer protein (CETP), a glycoprotein synthesized in the liver, facilitates the transfer of cholesteryl esters from high-density lipoprotein (HDL) particles to apolipoprotein B (apoB)-containing particles and boosts the transfer of triglycerides from apoB-containing particles to HDL particles [3]. Numerous research studies have demonstrated CETP is closely related to the development of coronary heart disease and atherosclerosis. A previous study showed that CETP expression induced oxidative stress and endoplasmic reticulum stress in endothelial cells, resulting in endothelial dysfunction and atherosclerosis [4]. Another existing study indicated CETP inhibition decreased major adverse cardiovascular events by lowering apoB levels [5]. A large body of evidence has suggested that CETP is an effective drug target for the treatment of cardiovascular diseases.

Ginsenoside Re (Re) is one of the protopanaxatriol-type saponins derived from ginseng, a well-known traditional Chinese herb widely used in treating various diseases, such as cardiovascular disease, nervous system disorders, diabetes mellitus, and cancer [6]. Re exhibits multiple pharmacological activities, including cardioprotective, neuroprotective, anti-diabetic, anti-inflammatory, anti-cancer, and antioxidant effects [7]. Moreover, Re could regulate cholesterol metabolism [8], reduce insulin resistance, and improve glucose uptake [9]. Previous *in vitro* and *in vivo* studies have shown that Re protects myocardial injury by regulating AMPK/TGF-β1/Smad2/3 and FAK/PI3K p110α/Akt pathways [10], inhibiting HIF-1α ubiquitination [11] and improving antioxidant and anti-inflammatory capacities [12,13]. Although the mechanism of its cardioprotective efficacy has been discussed, it has not been fully understood. This study was performed to observe the therapeutic effects of Re on isoproterenol (ISO)-induced cardiac hypertrophy *in vitro* and *in vivo* and to explore whether the underlying mechanism of Re in alleviating cardiac hypertrophy was associated with inhibition of CETP expression.

## Materials and methods

Reagents

**Figure 1:**
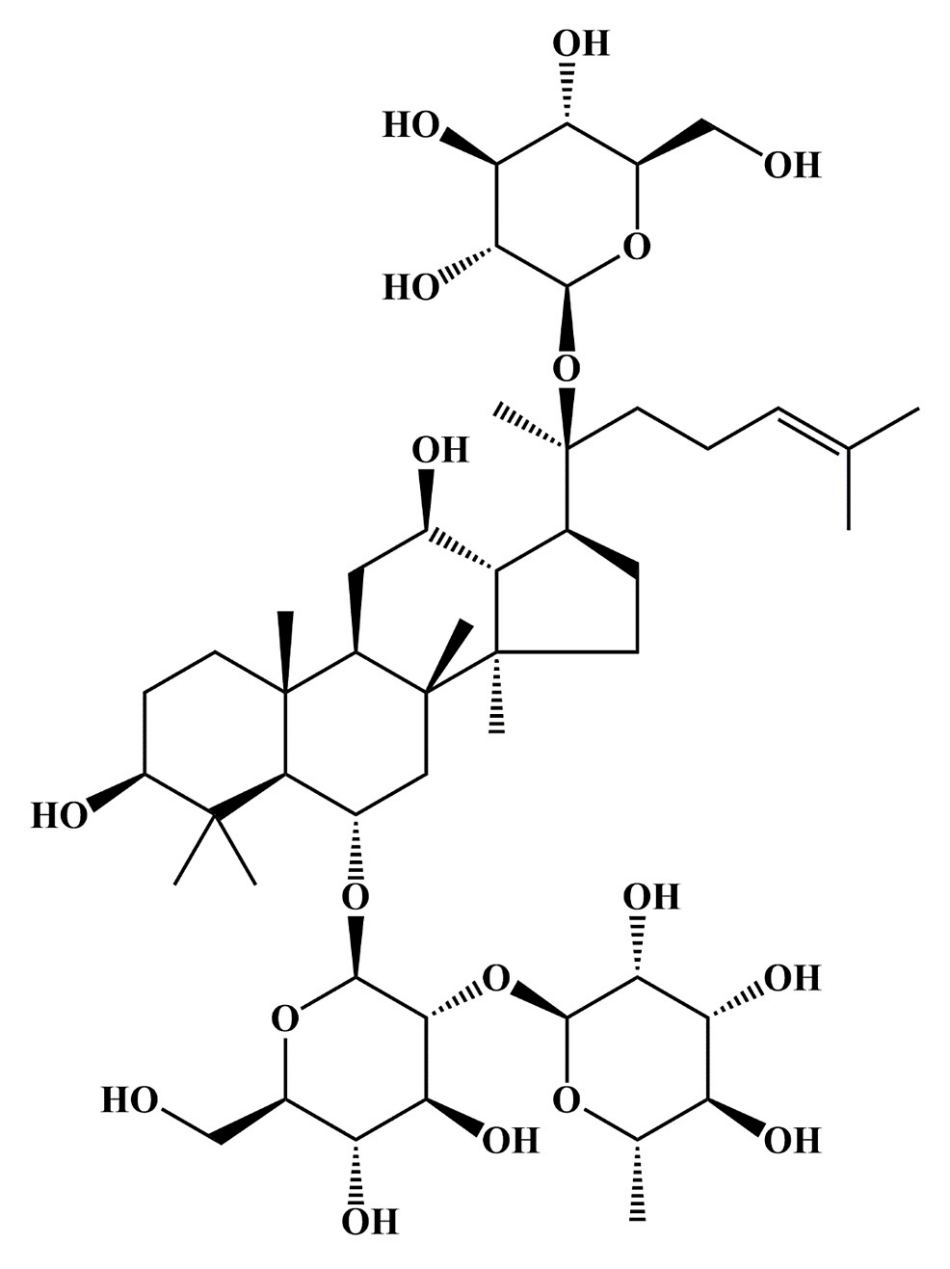
Chemical structure of ginsenoside Re.

Re (purity > 99.9%, 110754-202129) and ISO (purity > 99.9%, 100166-202205) for cell culture were obtained from the National Institutes for Food and Drug Control (Beijing, China). Re (purity > 95%, S27663) and ISO (purity ≥ 98%, S31064) for animal experiments were purchased from Shanghai Yuanye Bio-Technology Co., Ltd (Shanghai, China). The chemical structure of Re is depicted in Figure 1. Atorvastatin calcium tablets (ATC, 20 mg/tablet) were acquired from Pfizer Inc. (New York, NY). Anacetrapib (HY-12090) was provided by MedChemExpress (Monmouth Junction, NJ). PrimeScript™ RT reagent Kit with gDNA Eraser (Perfect Real Time) and TB Green® Premix Ex Taq™ II were obtained from Takara (Shiga, Japan). Anti-CETP antibody (ab19012) was purchased from Abcam (Cambridge, UK). β-actin antibody (sc-47778) was obtained from Santa Cruz Biotechnology Co., Ltd. (Dallas, TX). The bicinchoninic acid (BCA) protein assay kit and enhanced chemiluminescence assay kit were furnished by Beyotime Biotechnology (Shanghai, China). Enzyme-linked immunosorbent assay (ELISA) kit (MM-0382R2) was provided by Jiangsu Enzyme Immune Industrial Co., Ltd. (Jiangsu, China). Total cholesterol (TC, A111-1-1), triglyceride (TG, A110-1-1), low-density lipoprotein cholesterol (LDL-C, A113-1-1), and high-density lipoprotein cholesterol (HDL-C, A112-1-1) assay kits were purchased from Nanjing Jiancheng Bioengineering Institute Co., Ltd (Nanjing, China).

Animals

Male specific pathogen-free (SPF) grade Sprague-Dawley (SD) rats (n = 42, five to six weeks old, 200-250 g) were purchased from the Jiangxi University of Chinese Medicine (License ID: SYXK (Gan) 2018-0003, Nanchang, China). All rats were housed at room temperature of 20-22°C, relative humidity of 50%, and a 12-hour light-dark cycle, with free access to food and water. The experimental protocols were approved by the Animal Care and Use Committee of Jiangxi Science & Technology Normal University (approval number: Y202206) and conducted in accordance with the Guide for the Care and Use of Laboratory Animals (NIH Publication No.: 85-23, revised 1996).

In vivo experimental protocol

Rats were randomly assigned into the following seven groups (six rats per group): control, ISO, ISO+atorvastatin 20 mg/kg (ISO+ATC), ISO+Re 20 mg/kg (ISO+Re-L), ISO+Re 40 mg/kg (ISO+Re-H), atorvastatin 20 mg/kg (ATC) and Re 40 mg/kg (Re-H) groups. ISO (30 mg/kg) was dissolved in saline and intraperitoneally injected into rats thrice daily for 14 consecutive days to induce cardiac hypertrophy. All treatments with atorvastatin and Re were orally administered once daily for three days in advance until the end of the experiment. Atorvastatin, an HMG-CoA (3-hydroxy-3-methylglutaryl coenzyme A) reductase inhibitor, was used as a positive control drug. The dose of atorvastatin at 20 mg/kg as well as two different doses of Re (20 mg/kg and 40 mg/kg) were chosen based on previously published literature [14,15]. After the last treatment, all rats were overnight fasted, weighed, and then anesthetized with pentobarbital sodium (50 mg/kg, intraperitoneally). Blood and tissues were collected for the following measurements.

Gravimetric measurements

The heart tissues were washed and weighed to calculate the ratio of heart weight to body weight (HW/BW). In addition, the left ventricular free wall (LV) and right ventricular free wall (RV) were separated and weighed to calculate ratios of left ventricular weight to body weight (LVW/BW) and right ventricular weight to body weight (RVW/BW), respectively. All tissues were frozen in liquid nitrogen.

Analysis of serum lipids

The serum was separated by centrifugation at 2,000 rpm for 10 minutes at 4℃ and stored at -80℃. The contents of serum TC, TG, LDL-C, and HDL-C were detected by enzymatic colorimetric assay using commercial kits according to the manufacturer’s instructions.

Histological staining

Fresh left ventricular tissues were fixed in 4% paraformaldehyde solution for more than 24 hours, and then embedded in paraffin and cut into 5 µm thickness sections, which were subsequently stained by hematoxylin and eosin to evaluate overall pathological changes. Moreover, the cardiomyocyte cross-sectional area was measured by ImageJ software (National Institutes of Health, Bethesda, MD).

Cell culture and treatment

Rat H9c2 cardiomyocytes were obtained from the Cell Resource Center, Peking Union Medical College (Beijing, China). Cells were cultured in Dulbecco’s modified Eagle’s medium (Solarbio, Beijing, China) supplemented with 10% fetal bovine serum (Gemini, West Sacramento, CA) at 37°C in a humidified atmosphere of 5% CO_2_. Cells were inoculated in 35 mm culture dishes and allowed to grow to 80% confluence, then pretreated with Re or anacetrapib (a CETP inhibitor) for one hour, followed by stimulation with or without 10 µM ISO for 24 hours.

Cell viability assay

The effect of Re and anacetrapib on cardiomyocyte viability was observed by the MTT (3-(4,5-dimethylthiazol-2-yl)-2,5-diphenyltetrazolium bromide) assay described previously [16]. Briefly, cells were seeded in 96-well plates and exposed to various concentrations of Re (13.2, 26.4, 52.8, 105.6, and 211.2 µM) or anacetrapib (5, 10, 20, 40, and 80 µM) for 24 hours. Afterward, cells were incubated with 50 µL MTT (1 mg/mL) at 37˚C for four hours. The optical density values were measured at 490 nm.

Cell surface area measurement

Cells were imaged with an MI52-N inverted microscope (Guangzhou Mingmei Photoelectric Technology Co., Ltd., Guangzhou, China) equipped with an MShot Image Analysis System at 100× magnification. Cell surface area was measured by ImageJ software and calculated as the average value of 60 cells in each treatment group.

Cellular lipid level measurement

The levels of TC, TG, LDL-C, and HDL-C in cardiomyocytes were detected with commercial kits.

ELISA assay

The CETP level in cardiomyocytes was assessed using a commercial ELISA kit according to the manufacturer’s instructions.

Real-time polymerase chain reaction (RT-PCR)

Total RNA was extracted from either left ventricular tissues or cardiomyocytes using Trizol reagent according to the manufacturer’s instructions. A total of 1 μg of total RNA from each sample was reversely transcribed to cDNA at 37°C for 15 minutes and then at 85°C for five seconds. Polymerase chain reaction (PCR) was performed with TB Green® Premix Ex Taq™ II by CFX96™ Real-Time PCR (Bio-Rad, Hercules, CA) in conditions of pre-denaturation at 95°C for 30 seconds, followed by 40 cycles of denaturation at 95°C for 10 seconds, annealing at 60°C for 30 seconds, and extension at 72°C for 30 seconds. The mRNA relative expression level of atrial natriuretic peptide (ANP), β-myosin heavy chain (β-MHC), α-skeletal actin (α-SKA), and CETP was calculated by the standard curve method and normalized to 18S ribosomal ribonucleic acid (rRNA), respectively. The primer sequences were as follows: ANP, forward 5′-TCTCCATCACCAAGGGCTTC-3′, reverse 5′-TGACCTCATCTTCTACCGGC-3′; β-MHC, forward 5′-GAGTTCGGGCGAGTCAAAGA-3′, reverse 5′-AGCCTCTCGGTCATCTCCTT-3′; α-SKA, forward 5′-GAAGGACCTGTACGCCAACA-3′, reverse 5′-TCCACACTGAGTACTTGCGC-3′; CETP, forward 5′-ATTCCTCTTTCCACGCCCAG-3′, reverse 5′-AAGCTCTGGATGGACTCGGA-3′; and 18S rRNA, forward 5′-GGCCGTTCTTAGTTGGTGGA-3′, reverse 5′-TGAGCCAGTTCAGTGTAGCG-3′.

Western blot analysis

Total protein was extracted from left ventricular tissues or cardiomyocytes using radioimmunoprecipitation assay buffer containing 1 mM phenylmethylsulphonyl fluoride and the content was quantified by a BCA protein assay kit. Equal amounts of protein were separated by 10% SDS-PAGE (sodium dodecyl sulfate polyacrylamide gel electrophoresis) and then transferred onto the polyvinylidene difluoride (PVDF) membrane. After blocking with 5% bovine serum albumin for one hour, membranes were incubated with primary antibodies against CETP (1:2,000 dilution) and β-actin (1:500 dilution) at 4℃ overnight. Subsequently, membranes were incubated with secondary antibodies for 30 minutes at room temperature. Bands were visualized by an enhanced chemiluminescence assay kit and the intensity was determined using ImageJ software.

Statistical analysis

All data were expressed as means ± standard error of the mean (SEM). Statistical significance was analyzed using one-way ANOVA, followed by Tukey's multiple comparisons test through GraphPad Prism 8.0 software (GraphPad Software, San Diego, CA). P < 0.05 was considered statistically significant.

## Results

Re attenuated ISO-induced cardiac hypertrophy in vivo

**Figure 2:**
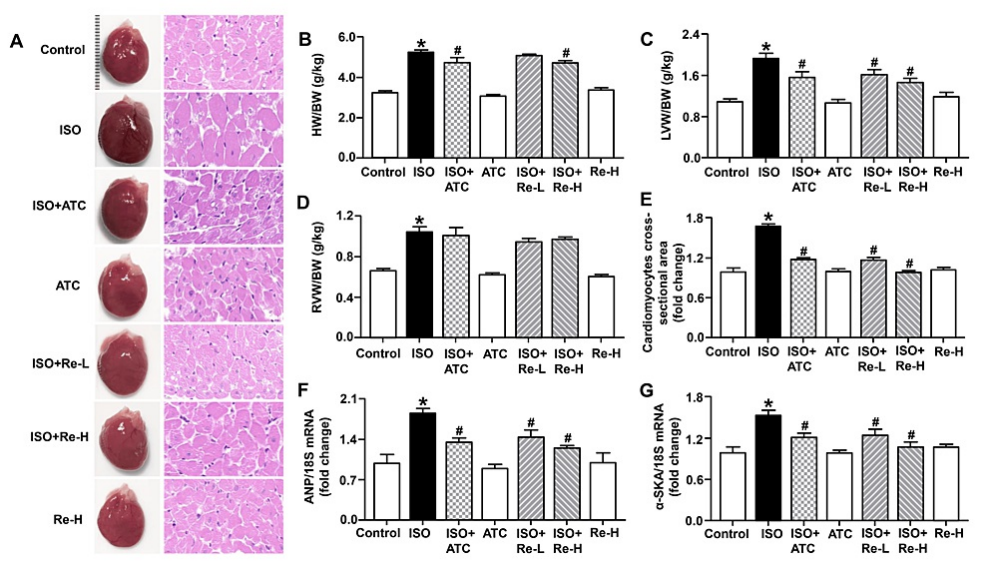
Ginsenoside Re attenuated isoproterenol-induced cardiac hypertrophy in rats. (A) Representative heart morphological photographs and representative images of left ventricular tissue sections stained with hematoxylin and eosin (magnification, ×630). (B-D) Ratios of HW/BW, LVW/BW, and RVW/BW were determined (n = 6). (E) Cardiomyocytes cross-sectional area based on morphological changes in left ventricular tissue sections stained with hematoxylin and eosin was measured (n = 3). The mRNA expression of ANP (F) and α-SKA (G) (n = 6) in left ventricular tissue was quantified, respectively. Data are shown as means ± SEM. * P < 0.05 vs. control group; ^# ^P < 0.05 vs. ISO group. ATC, atorvastatin; ISO, isoproterenol; HW, heart weight; BW, body weight; LVW, left ventricle weight; RVW, right ventricle weight; ANP, atrial natriuretic peptide; α-SKA, α-skeletal actin; SEM, standard error of the mean.

To investigate the effect of Re on cardiac hypertrophy *in vivo*, we established an ISO-induced cardiac hypertrophy model in rats treated with Re at doses of 20 and 40 mg/kg or atorvastatin 20 mg/kg. As shown in Figures 2A-2E, compared with the control group, rats treated with ISO presented an obvious hypertrophic response, as indicated by increases in heart size, ratios of HW/BW, LVW/BW, and RVW/BW, and cardiomyocytes cross-sectional area. Treatments with 40 mg/kg Re and 20 mg/kg atorvastatin significantly reduced ratios of HW/BW and LVW/BW, as well as cardiomyocytes cross-sectional area. In addition, 20 mg/kg Re markedly decreased the LVW/BW ratio and cardiomyocytes cross-sectional area and had a tendency to reduce the HW/BW ratio. Both Re and atorvastatin showed no obvious effects on the RVW/BW ratio. Moreover, a significant downregulation of mRNA levels of hypertrophy-related fetal genes ANP and α-SKA was observed in ISO-induced rats treated with both Re and atorvastatin (Figures 2F, 2G). Collectively, these data demonstrated that Re treatment significantly improved ISO-induced cardiac hypertrophy.

Re improved lipid abnormality in ISO-induced rats

**Figure 3:**
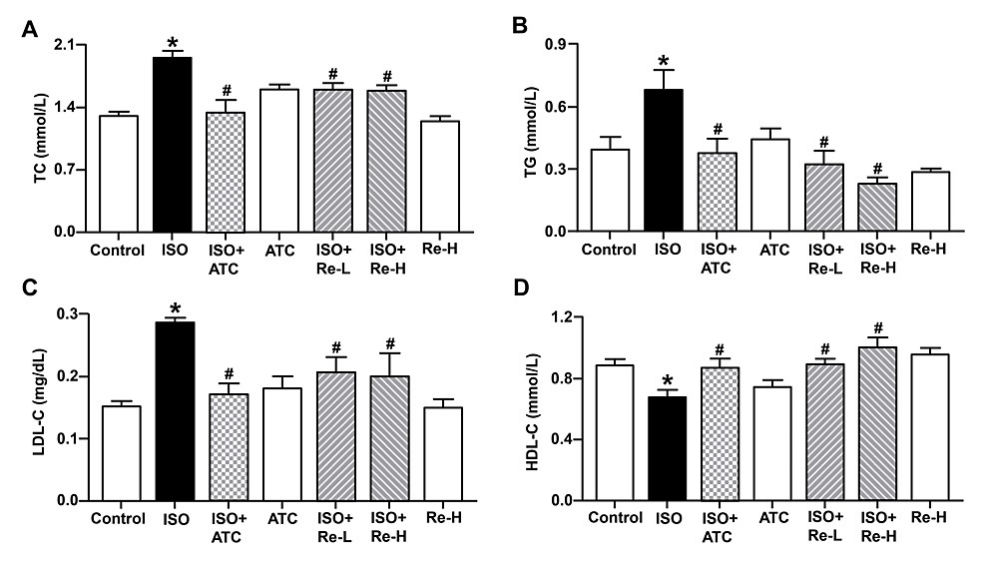
Effects of ginsenoside Re on serum TC (A), TG (B), LDL-C (C), and HDL-C (D) in isoproterenol-induced cardiac hypertrophy rats. Data are shown as means ± SEM (n = 6). * P < 0.05 vs. control group; ^# ^P < 0.05 vs. ISO group. ATC, atorvastatin; ISO, isoproterenol; TC, total cholesterol; TG, triglyceride; LDL-C, low-density lipoprotein cholesterol; HDL-C, high-density lipoprotein cholesterol; SEM, standard error of the mean.

Dyslipidemia is one of the most important independent risk factors for cardiovascular disease. We next analyzed the blood lipid levels in rats. As shown in Figure 3, compared with the control group, ISO induced increases in the levels of serum TC, TG, and LDL-C, as well as a decrease in the content of serum HDL-C; however, these characteristic changes were reversed by treatments with Re and atorvastatin. These results suggest that Re could alleviate ISO-induced disorders of lipid metabolism in rats.

Re suppressed ISO-induced cardiomyocyte hypertrophy in vitro

**Figure 4:**
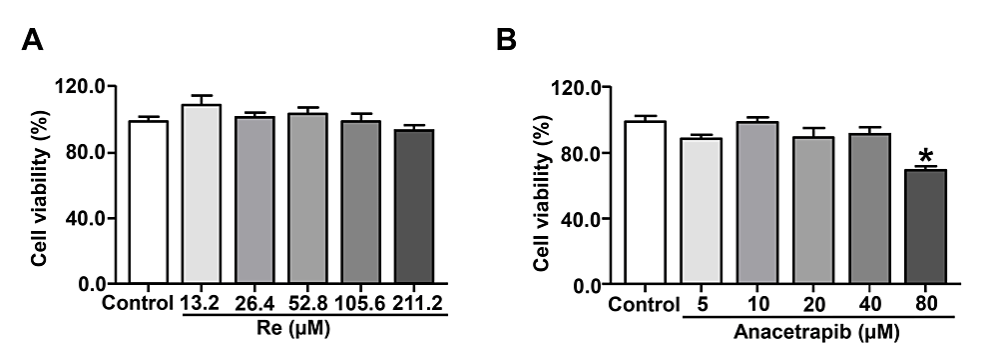
Effects of ginsenoside Re (A) and anacetrapib (B) on H9c2 cardiomyocyte viability. H9c2 cardiomyocytes were exposed to Re (13.2, 26.4, 52.8, 105.6, and 211.2 µM) or anacetrapib (5, 10, 20, 40, and 80 µM) for 24 hours. Cell viability was determined using the MTT assay. Data are shown as means ± SEM (n = 5). * P < 0.05 vs. control group. Re, ginsenoside Re; MTT, 3-(4,5-dimethylthiazol-2-yl)-2,5-diphenyltetrazolium bromide; SEM, standard error of the mean.

We next explored the effect of Re on ISO-induced cardiomyocyte hypertrophy *in vitro*. We performed a cell viability assay by the MTT method and the data showed that Re at the concentration range of 13.2-211.2 µM showed no toxicity to H9c2 cardiomyocytes (Figure 4A). In addition, anacetrapib at the concentration series of 5, 10, 20, and 40 μM did not show any obvious change in cell survival; however, anacetrapib 80 µM exhibited a significant cytotoxic effect (Figure 4B). Therefore, we used 105.6 µM Re and 40 µM anacetrapib in further experiments.

**Figure 5:**
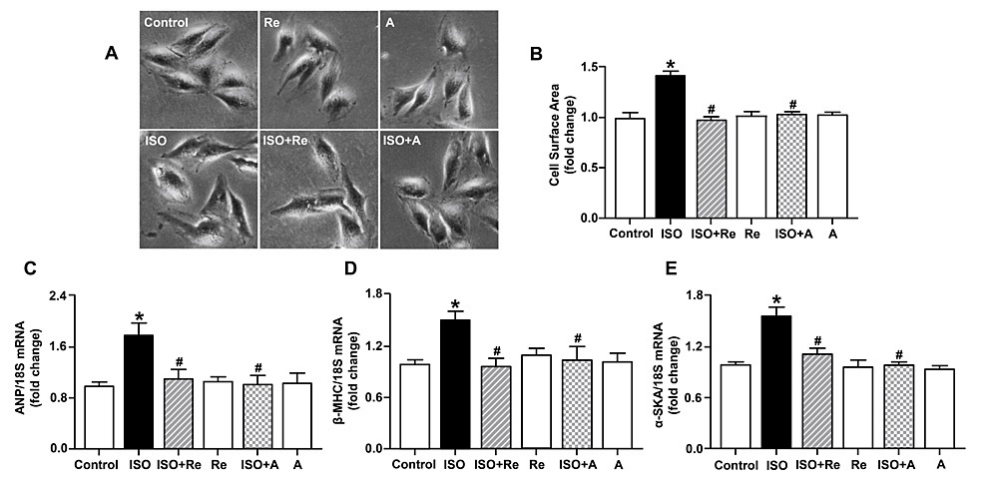
Ginsenoside Re inhibited isoproterenol-induced cardiomyocyte hypertrophy. (A) Representative microscopy images of each group (100×). Re and anacetrapib-inhibited isoproterenol induced significant increases in cell surface area (B) as well as mRNA levels of ANP (C), β-MHC (D), and α-SKA (E). Data are shown as means ± SEM (n = 5). * P < 0.05 vs. control group; ^# ^P < 0.05 vs. ISO group. A, anacetrapib; ISO, isoproterenol; ANP, atrial natriuretic peptide; β-MHC, β-myosin heavy chain; α-SKA, α-skeletal actin; SEM, standard error of the mean.

Cardiomyocytes exposed to 10 µM ISO for 24 hours exhibited significant hypertrophy as characterized by increased cell surface area (Figures 5A, 5B) and upregulation of mRNA expression of fetal genes, including ANP, β-MHC, and α-SKA, compared with control cells (Figures 5C-5E); however, treatment with both 105.6 µM Re and 40 µM anacetrapib significantly depressed these above changes. Our results suggest that Re and anacetrapib have similar inhibitory effects on ISO-induced cardiomyocytes hypertrophy.

Re ameliorated lipid abnormality in ISO-induced cardiomyocytes

**Figure 6:**
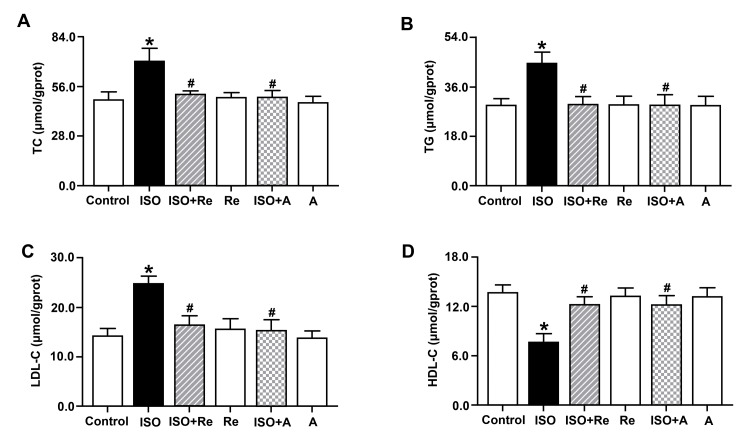
Effects of ginsenoside Re on TC (A), TG (B), LDL-C (C), and HDL-C (D) in isoproterenol-induced cardiomyocytes. Data are shown as means ± SEM (n = 6). * P < 0.05 vs. control group; ^# ^P < 0.05 vs. ISO group. A, anacetrapib; ISO, isoproterenol; TC, total cholesterol; TG, triglyceride; LDL-C, low-density lipoprotein cholesterol; HDL-C, high-density lipoprotein cholesterol; SEM, standard error of the mean.

As shown in Figure 6, TC, TG, and LDL-C levels were significantly increased and HDL-C level was obviously decreased in cardiomyocytes exposed to ISO stimulation, but these changes were significantly inhibited by both 105.6 µM Re and 40 µM anacetrapib, which is similar to our *in vivo* data.

Re downregulated CETP expression in ISO-induced cardiac hypertrophy

**Figure 7:**
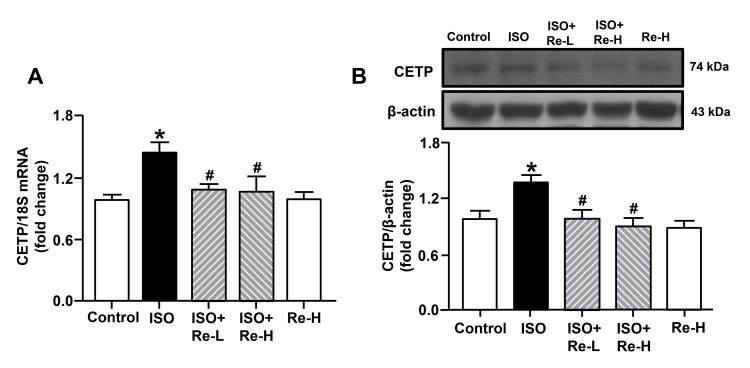
Effects of ginsenoside Re on CETP expression in left ventricular tissues of cardiac hypertrophy rats. (A) The mRNA level of CETP was observed. (B) Representative western blots and quantification of CETP. Data are shown as means ± SEM (n = 6). * P < 0.05 vs. control group; ^# ^P < 0.05 vs. ISO group. ISO, isoproterenol; CETP, cholesteryl ester transfer protein; SEM, standard error of the mean.

**Figure 8:**
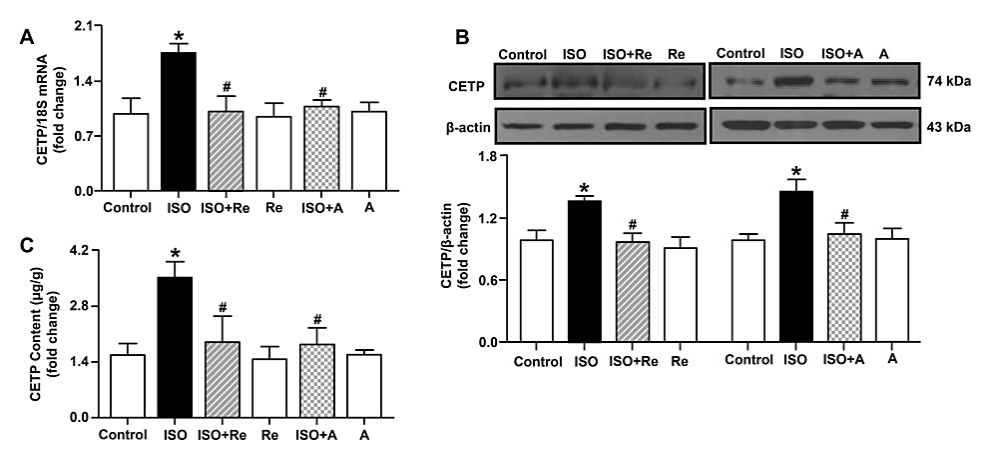
Effects of ginsenoside Re on the expression of CETP in cardiomyocytes. The mRNA level (A) and protein expression (B) of CETP (n = 5) as well as CETP content in cardiomyocytes (C) (n = 4). Data are shown as means ± SEM. * P < 0.05 vs. control group; ^# ^P < 0.05 vs. ISO group. A, anacetrapib; ISO, isoproterenol; CETP, cholesteryl ester transfer protein; SEM, standard error of the mean.

CETP has been considered a therapeutic target for cardiovascular diseases for decades. We further investigated whether Re could improve cardiac hypertrophy by regulating CETP expression by RT-PCR, western blot, and ELISA assays. Our in vivo experiments showed that Re (20 and 40 mg/kg) inhibited ISO-induced upregulations of CETP mRNA level and protein expression (Figure 7). Moreover, our *in vitro* data also demonstrated treatments with both 105.6 µM Re and 40 µM anacetrapib significantly suppressed mRNA and protein levels of CETP as well as CETP content in ISO-induced cardiomyocytes hypertrophy (Figure 8). Thus, the effect of Re in alleviating ISO-induced cardiac hypertrophy may be associated with suppression of CETP.

## Discussion

Pathological cardiac hypertrophy is a process of abnormal myocardium remodeling that initially occurs in response to numerous stimuli, such as ischemia and hypoxia, pressure or volume overload, hormones, etc., and eventually progresses to heart failure. It has emerged as an independent risk factor for increased morbidity and mortality of heart failure [17]. Currently, a variety of *in vivo* and *in vitro* experimental approaches to mimic cardiac hypertrophy have been developed to search for new therapeutic strategies, mainly including neurohormonal activation via inducers, such as ISO, angiotensin II, phenylephrine, and endothelin-1, as well as surgical procedures, for instance, transverse aortic constriction and coronary artery ligation [18].

ISO is a non-selective β_1_ and β_2_ adrenergic receptor agonist and has been widely used to induce cardiac hypertrophy in mice and rats. It has been reported in the literature that consecutive administration of ISO at a certain dose through mini-osmotic pump implantation, intraperitoneal, or subcutaneous injection over two to four weeks could generate a hypertrophic response in most rats [19-21]. In this study, intraperitoneal injection of ISO at 30 mg/kg thrice daily for 14 consecutive days resulted in increases in cross-sectional cardiomyocytes area, ratios of HW/BW, LVW/BW, and RVW/BW, as well as reactivation of fetal genes; however, treatment with Re ameliorated most of these hypertrophic responses, which is in line with a previous study [15]. Consistent with the *in vivo* results, Re treatment markedly blunted ISO-induced cardiomyocytes hypertrophy, as indicated by a decreased cell surface area and downregulation of fetal genes.

It has been reported that ISO could enhance heart rate and contractility and exaggerate myocardial oxygen consumption, which perturbs the physiological oxidant/antioxidant balance, increasing lipid peroxidation and depleting antioxidant enzymes [22]. ISO-administrated rats showed increases in the level of plasma TC, TG, phospholipids, free fatty acids, very-low-density lipoprotein cholesterol (VLDL-C), and LDL-C, and a decrease in HDL-C [23]. In our *in vivo* and *in vitro* experiments, we found that Re ameliorated ISO-induced increases in TC, TG, and LDL-C levels, as well as a decrease in HDL-C level, which is similar to the effect of positive control atorvastatin or anacetrapib, respectively. In agreement with the present results, previous research studies have shown Re effectively decreased the levels of TG, TC, LDL-C, and lipoprotein (a), and increased HDL-C levels in diabetic rats or mice models [24,25]. Existing evidence has demonstrated that Re could lower blood glucose and lipid levels via activation of AMP-activated protein kinase [26]; moreover, Re modulates cholesterol metabolism via upregulation of CYP8B1 mRNA level, leading to alteration of the biosynthesis and disposition of bile acids [8]. The results from this study and previous reports suggest that Re may have the potential to regulate lipid metabolism, particularly cholesterol metabolism.

It is well known that CETP is a key enzyme involved in cholesterol metabolism and transportation, which transfers cholesteryl esters from HDL to low-density lipoprotein (LDL) in exchange for triglycerides. In the past decades, CETP has been considered to be one of the most ideal targets for lipid-lowering, and inhibition of CETP could improve cardiovascular disease outcomes [27,28]. Earlier research studies have shown that multiple CETP inhibitors, including torcetrapib, dalcetrapib, evacetrapib, and anacetrapib, were designed to raise plasma HDL-C levels; however, most of these compounds except for anacetrapib failed to reduce the risk for cardiovascular disease due to compound-specific reasons [29]. Nevertheless, these failures have also promoted a shift in the research focus of CETP inhibitors, from raising HDL-C to lowering apoB-containing lipoproteins [3,5]. Emerging evidence revealed that obicetrapib, one of the newest generation CETP inhibitors, was developed to lower LDL-C and apoB, and has shown its potential in reducing cardiovascular disease risk in phase 2 clinical trials [30].

To further explore the mechanism of Re in improving cardiac hypertrophy, we investigated the influence of Re on the CETP expression *in vivo* and *in vitro* through RT-PCR, western blot, and ELISA assays. Our data showed that treatment with Re suppressed CETP mRNA level and protein expression as well as CETP content. Of note, the *in vitro* results showed that the inhibitory effect of Re on CETP is comparable to that of anacetrapib. These results suggest that the cardioprotective effects of Re is associated with its inhibition of CETP.

Limitations

It is important to note that current research has some limitations. For instance, CETP activity in both left ventricular tissues of rats and H9c2 cardiomyocytes was not measured, which could have provided further insights into the role of Re on ISO-induced cardiac hypertrophy. In addition, the use of rats as a model organism for the *in vivo* experiments in this study might not be ideal. The metabolic processes in rats are different from those in humans, which can lead to varying drug effects. Nevertheless, the findings of this study will provide insights for future studies investigating the effects of Re on CETP expression in different animal tissues.

## Conclusions

In the present study, we demonstrated that treatment with Re inhibited ISO-induced cardiac hypertrophy *in vivo* and *in vitro*, accompanied by decreases in TC, TG, and LDL-C levels, and an increase in HDL-C levels. The mechanism that might be involved is that Re inhibited the mRNA level and protein expression of CETP. Re may become a potential therapeutic agent for cardiovascular diseases.
